# Erythropoietin therapy in a case of neonatal anemia after exposure to natalizumab throughout pregnancy

**DOI:** 10.1186/s13052-021-01025-4

**Published:** 2021-03-23

**Authors:** Elisabetta Godano, Fabio Barra, Alessandra Allodi, Antonella Ferraiolo, Alice Laroni, Giovanni Novi, Giovanni Luigi Mancardi, Claudio Gustavino, Cesare Arioni

**Affiliations:** 1 Neonatology Unit, IRCCS Ospedale Policlinico San Martino, Genoa, Italy; 2 Academic Unit of Obstetrics and Gynaecology, IRCCS Ospedale Policlinico San Martino, Largo R. Benzi 10, 16132 Genoa, Italy; 3 Unit of Obstetrics and Gynaecology, IRCCS Ospedale Policlinico San Martino, Genoa, Italy; 4 Academic Unit of Neurology, IRCCS Ospedale Policlinico San Martino, Genoa, Italy; 5 Department of Neuroscience, Rehabilitation, Ophthalmology, Genetics, Maternal and Child University of Genova, Genoa, Italy; 6 IRCCS ICS Maugeri, Pavia, Italy

**Keywords:** Natalizumab, Anemia, Pregnancy, Erythropoietin, Multiple sclerosis

## Abstract

**Background:**

Natalizumab is a monoclonal antibody approved for the treatment of patients with relapsing-remitting multiple sclerosis. According to the current clinical recommendations, its use during pregnancy should be carefully evaluated only in women with highly active disease who plan a pregnancy or have an unplanned pregnancy, after accurate counseling about eventual maternal disease relapse due to therapy suspension.

**Case presentation:**

This brief case report describes a case of documented anemia that we observed in a newborn whose mother with relapsing-remitting multiple sclerosis was treated with an extended dosing protocol of natalizumab throughout pregnancy. The newborn received the infusion of erythropoietin every seven days from the fortieth day of life; subsequently, the status of anemia underwent clinical resolution.

**Conclusions:**

This case report confirmed that natalizumab can cause disorders of hematopoiesis, including anemia, thrombocytopenia, or pancytopenia, in newborns of patients treated during pregnancy. A multidisciplinary team, including experienced pediatricians and pediatric hematologists, has a critical role in managing newborns delivered by women, being treated with natalizumab for treating relapsing-remitting multiple sclerosis during pregnancy.

## Background

Natalizumab is a monoclonal antibody approved for the treatment of patients with relapsing-remitting multiple sclerosis (MS) [1]. It is a humanized monoclonal antibody (class IgG4) that binds to the alfa-4 integrin on the surface of lymphocytes and inhibits peripheral blood lymphocyte migrating to the central nervous system (CNS), involved in the cerebral inflammatory microenvironment occurring in MS [2]. Natalizumab four-weekly intravenous infusion is well tolerated in the short-term administration [3].

Transport of maternal antibodies of class IgG (any subclass) via the placental barrier starts in the second trimester, and it increases as pregnancy progresses [4], including several monoclonal antibodies therapeutic given for therapeutic purpose [5]. Placental transport of natalizumab was first described in 2014 [6].

As the fertile period of women often coincides with the period in which MS is diagnosed, in recent years many questions have been raised about the safety of this drug during pregnancy [7]. At the moment, natalizumab is classified as a pregnancy category C drug; this reflects the lack of adequate and well-controlled studies in humans [8]. A review of the literature indicated that the spontaneous abortion rate of MS women having received natalizumab during pregnancy is similar to that of the general population [7]; until now, different case reports have documented favorable pregnancy outcomes in women receiving natalizumab [9, 10].

It is known that newborns of mothers exposed to natalizumab during the third trimester can develop disorders of hematopoiesis such as anemia, thrombocytopenia, or pancytopenia [11]. These hematological disorders tend to be asymptomatic.

This case report aims to describe a case of neonatal anemia that we observed in a newborn whose mother received natalizumab treatment during pregnancy through an extended interval dosing (EID) protocol; the newborn was treated with infusion of erythropoietin every seven days from the fortieth day of life in total well-being.

## Case presentation

We report a case of a baby born in May 2020 at full term in a pregnant woman affected by relapsing-remitting MS, which was diagnosed in 2009 and has been treated with natalizumab since 2011. This treatment was temporarily interrupted and replaced with glatiramer acetate, an immunomodulatory drug, during the previous pregnancy in 2016; this change of treatment led to disease relapse immediately after the delivery, which was controlled restarting natalizumab in the puerperium; therefore, after a joint discussion between physicians and the patient, the pregnant woman was advised to continue natalizumab treatment during this second pregnancy, extending the interval among doses to six weeks [12]. Considering the worldwide emergency caused by SARS-CoV-2, maternal nasopharyngeal swab and blood sample were performed before the hospitalization scheduled for childbirth and both resulted negative for detecting the virus.

The baby was born by an elective cesarean section done at 37 + 5/7 weeks of gestation, which was performed because of the presence of a maternal brain parietal cavernoma. The baby did not undergo delayed cord clamping at birth. The baby’s weight was 2915 g, her length was 48 cm, and her cranial circumference was 34 cm (anthropometric parameters suitable for gestational age). The APGAR score at 1 and 5 min was 9/10; globally, the newborn underwent a regular adaptation to extrauterine life. Considering the data from the current literature about the hematological effects due to maternal therapy with natalizumab during pregnancy [7], we performed blood examination to the newborn after birth: as expected, low-moderate anemia (hemoglobin: 10.9 g/dl; hematocrit 34.1%, MCH 34.4 pg, MCHC 319 g/L) with standard value of leukocytes (17.84 × 10^9^/L) and platelets (196 × 10^9^/L) was described. No other specific investigations were performed to analyze other possible causes of anemia. During the first three days of life, the newborn had regular vital parameters and clinical conditions; moreover, it started to be fed with infant formula because of the plan to continue natalizumab treatment after delivery. Her weight loss was physiological and routinely neonatal tests, such as otoacoustic emission, direct Coomb’s test, and red reflex, were normal.

The newborn was treated with the administration of oral vitamin D (400 UI/ day), folic acid (0.11 mg/day), and chelated bisglycinate iron (0.75 mg/kg/ day). In accord with our local guidelines, the newborn was discharged after three days of hospitalization.

A 7- and 14-day follow-up of the newborn showed an adequate weight improvement. We performed blood samples each 7–14 days in the first month, showing a progressive worsening of the anemia. When, approximately at 40 days of life, the hemoglobin was 8 g/dl and the hematocrit was 23.5%, in multidisciplinary accord to the hematologists, we decided to start therapy with erythropoietin (EPO) at the dosage of 450 UI/kg every seven days. From the beginning of this therapy, the clinical condition of the baby was routinely evaluated by performing blood samples every three weeks and then monthly, showing a progressive increase in blood cell parameters.

When the baby was four months old, the hemoglobin was 12 g/dl and the hematocrit was 37.4%; thus, the EPO was interrupted (a total of 11 doses had been administered). One month after, the hemoglobin and the hematocrit were 12.1 g/dl and 37.9%, respectively; the clinical condition and weight of the newborn remained regular. Currently, we are doing monthly phone contacts with the newborn’s family in order to check the health status of the baby; furthermore, as the newborn’s family lives in another Italian region, clinical visits are being done by the general pediatrician. At one year from the delivery, a complete pediatric assessment with blood count will be performed at our institution.

## Discussion

Natalizumab is a highly effective drug employed for treating relapsing-remitting MS [13]. Its use during pregnancy is controversial: the ECTRIMS/EAN guidelines suggested considering the use of natalizumab in women with highly active evolution who plan a pregnancy or have an unplanned pregnancy, after a joint discussion between physicians and patients about possible risks under this chronic therapy [14].

It has been described that natalizumab can be detected in the umbilical cord blood, confirming that it crosses the blood–placental barrier; this can suggest a direct pharmacological impact on the fetus. To this purpose, it has been reported mild thrombocytopenia and anemia in the newborns delivered after 13 pregnancies with third-trimester exposure to natalizumab [11]. The occurrence of hematological alterations in the baby seems to be associated with natalizumab treatment during the third trimester [7, 8]. The mechanism underlying anemia could be explained by the presence of α4β1 (VLA-4) integrin, the target of natalizumab, on the surface of fetal blood cell precursors. In fact, differentiation of blood cell precursors can be inhibited by the presence of a monoclonal antibody that binds VLA-4 [15]. However, this effect has not been shown in all pregnancies [16].

Regarding our experience, we observed that the use of natalizumab during the whole period of pregnancy seems not to cause serious affections to the newborn. In this case, we decided to administer natalizumab by an EID protocol, which is broadly adopted for treating patients with MS with positive serum serology for JC virus to decrease the risk of progressive multifocal leukoencephalopathy (PML) [17]; the last dose of natalizumab was administered to the mother three weeks before delivery. From the current literature, it has been reported that in mother-infant serum pairs, natalizumab is only detectable when the last infusion was given less than 75 days before delivery; moreover, mothers, whose newborns develop hematological abnormalities, tend to receive last natalizumab infusion 3–1 months before delivery [8].

Natalizumab is also detectable in the majority of the breast milk samples of patients treated with natalizumab: the concentration level of free natalizumab in breast milk is significantly correlated to the time since the last natalizumab infusion has been given [18]; as such, breastfeeding is contraindicated during treatment with natalizumab.

Anemia is a physiological phenomenon in newborns [19]: it typically occurs at two-three months of life and hemoglobin tends to reach a nadir value of 9–11 g/dl and a spontaneous increase with a normal value at four-six months of life. Despite this, it is of extreme importance to monitor and eventually treat the clinical onset of anemia related to drugs characterized by systemic exposure like natalizumab. In our case report, the baby had always regular clinical conditions, gaining weight by being fed with infant formula; the newborn developed exclusively anemia. Despite the strong suspicion of anemia due to the exposure to natalizumab, the baby did not undergo delayed cord clamping at birth; in fact, at our institution, this procedure is only considered after eutocic delivery. In the future, it could be of interest to study the impact of delayed cord clamping following cesarean sections for preventing anemia in this setting; however, it should be only performed whenever the clinical conditions of either fetus or mother are stable at the time of birth [20].

We decided to perform a strict neonatal follow-up and afterward to start the EPO therapy to support the newborn bone marrow production. In agreement with the hematologists, EPO was administered at 450–500 UI/kg each 7 days. By performing seriates blood samples, we observed the nadir of hemoglobin after 40 days of life; the value of hemoglobin increased progressively after starting EPO administration.

No specific investigations were done to analyse other causes of anemia because its onset was expected after natalizumab treatment, as reported in the current literature [11]; moreover, there were not risk obstetrics factors sustaining the anemia onset in the newborn; lastly, the response to EPO therapy increases the evidence that the anemia was subsequent to natalizumab therapy and seems to exclude several other etiologies.

Overall, the critical importance of performing accurate planning of the delivery in patients receiving natalizumab during pregnancy should be underscored; the delivery should be planned and managed by a multidisciplinary team; this is necessary to adequately take charge of the baby and to perform an accurate follow up monitoring the conditions of the newborn for at least 3–4 months after birth; in fact, it should be considered that hematological alterations can even become evident few months after the last natalizumab infusion to the mother [21].

In conclusion, current clinical data on the safety of the use of natalizumab during pregnancy remain limited, despite being reassuring. This case report describes the neonatal clinical presentation of a newborn delivered by a woman under chronic treatment with natalizumab during pregnancy. We are aware that our study cannot support the unconditionally and safe use of this drug during the gestational period. Our purpose is to underscore the importance of adequately managing newborns after natalizumab treatment during the maternal gestational period.

## Data Availability

Data sharing is not applicable to this article as no datasets were generated or analysed during the current study.
